# Association of PD-L1 Expression on Tumor and Immune Cells with Survival in Recurrent or Metastatic Head and Neck Squamous Cell Carcinoma and Assay Validation

**DOI:** 10.1158/2767-9764.CRC-21-0032

**Published:** 2022-01-20

**Authors:** Sophie Wildsmith, Jiabu Ye, April Franks, Giovanni Melillo, Jon Armstrong, Jessica Whiteley, Karina Schnittker, Fangru Lian, Bryan Roland, Constantine Sabalos, Payam Ahmadi, Jerome Fayette, Caroline Even, Ricard Mesía, Lillian L. Siu, Dan P. Zandberg, Jill Walker

**Affiliations:** 1 Precision Medicine, R&D Oncology, AstraZeneca, Cambridge, United Kingdom.; 2 Statistics, AstraZeneca, Gaithersburg, Maryland.; 3 Global Medical Affairs, AstraZeneca, Gaithersburg, Maryland.; 4 Statistics, AstraZeneca, Macclesfield, United Kingdom.; 5 Companion Diagnostics Development, Ventana Medical Systems, Tucson, Arizona.; 6 Companion Diagnostics Pathology, Ventana Medical Systems, Tucson, Arizona.; 7 Companion Diagnostics Project Lead, Ventana Medical Systems, Tucson, Arizona.; 8 Regulatory Affairs, Companion Diagnostics, Ventana Medical Systems, Tucson, Arizona.; 9 Biometrics, Ventana Medical Systems, Tucson, Arizona.; 10 Department of Medical Oncology, Centre Léon Bérard, Lyon, France.; 11 Head and Neck Oncology Department, CLCC Institut Gustave Roussy, Paris, France.; 12 Medical Oncology Department, Institut Català d'Oncologia Badalona, B-ARGO group, IGTP, Barcelona, Catalonia, Spain.; 13 Division of Medical Oncology and Hematology, Princess Margaret Cancer Centre, Toronto, Ontario, Canada.; 14 Department of Hematology/Oncology, UPMC Hillman Cancer Center, Pittsburgh, Pennsylvania.

## Abstract

Programmed cell death ligand-1 (PD-L1), expressed on both tumor cells (TC) and tumor-associated immune cells (IC), has been shown to be a useful biomarker and predictive of response to anti-PD-L1 agents in certain tumor types. In recurrent or metastatic head and neck squamous cell carcinoma (R/M HNSCC), there is a growing interest in the role of PD-L1 expression on ICs, as well as TCs, for predicting response to immune checkpoint inhibitors. Using pooled data from the phase II HAWK and CONDOR studies, we investigated the association of baseline PD-L1 expression with durvalumab efficacy in patients with R/M HNSCC. To determine an optimal PD-L1 cut-off point for predicting survival, we assessed PD-L1 expression levels at different TC and IC cut-off points in patients treated with durvalumab. Longer survival was associated with higher TC membrane PD-L1 expression and IC staining. When the combined TC/IC algorithm was applied, a cut-off point for PD-L1 expression of ≥50% on TCs or ≥25% on ICs (TC ≥ 50%/IC ≥ 25%) showed a higher objective response rate (17.2% vs. 8.8%), longer median progression-free survival (2.8 vs. 1.9 months), and longer median overall survival (8.4 vs. 5.4 months) in the PD-L1–high versus PD-L1–low/negative patient populations, respectively. A scoring algorithm combining PD-L1 expression on TCs and ICs using the cut-off point TC ≥ 50%/IC ≥ 25% was optimal for identifying patients with HNSCC most likely to benefit from durvalumab treatment. The new algorithm is robust and can be reproducibly scored by trained pathologists.

**Significance:**

A novel algorithm for PD-L1 expression using the cut-off point TC ≥ 50%/IC ≥ 25% is robust for identifying patients with HNSCC most likely to benefit from durvalumab treatment and can be reproducibly scored by trained pathologists.

## Introduction

Appropriate patient selection and biomarker testing are key to guiding the use of immune checkpoint inhibitors (ICI) to improve the care of patients with recurrent or metastatic head and neck squamous cell carcinoma (R/M HNSCC; ref. 1). Programmed cell death ligand-1 (PD-L1) is the most widely used biomarker in clinical practice and has been associated with improved response and prolonged survival with ICIs in HNSCC (2, 3). Currently, various PD-L1 IHC diagnostic assays are utilized to evaluate PD-L1 as a biomarker in various types of tumors, with different antibodies, thresholds (cut-off points), and algorithms for classifying samples as PD-L1–high or PD-L1–low/negative (4–6).

Assays required for the safe and efficacious use of therapeutics must be fully validated and approved as diagnostic devices. The testing platform is specific to the diagnostic developer, while the algorithms and cut-off points are determined on the basis of pharmaceutical efficacy data. In early clinical development, small patient numbers pose a key challenge for developing an assay that best selects patients who may benefit from ICI therapy. Currently, limited clinical outcome data are available and usually include objective response rate (ORR) and progression-free survival (PFS) with limited follow-up. Meta-analyses indicate that ORR is not a good predictor of overall survival (OS) outcomes for ICIs (7–9). Hence, the optimal algorithm and cut-off point for immunotherapy is reviewed when new data are available and often revised. For example, in the CheckMate 141 study of nivolumab in platinum-refractory R/M HNSCC, PD-L1 was assessed at expression levels of ≥1%, ≥5%, and ≥10% in a minimum of 100 tumor cells (TC) that could be evaluated (10). On the basis of the outcome of this study, a diagnostic was developed with a cut-off point of TC1%. Early studies of pembrolizumab in a similar population (KEYNOTE-012) also used a cut-off point of TC1% (11). Using data from 132 patients in this study, it was demonstrated that TC PD-L1 expression alone was not significantly correlated with response. Furthermore, including PD-L1 expression on immune cells (IC) significantly contributed to the predictive value (12). The new combined scoring method was incorporated in study endpoints for subsequent pembrolizumab trials in R/M HNSCC (2, 13).

The original PD-L1 cut-off point for durvalumab, an anti-PD-L1 antibody, was determined using ORR data from 50 patients with R/M HNSCC enrolled in the phase I/II study (Study 1108; refs. 4, 14). PD-L1 expression was assessed on TCs using the VENTANA PD-L1 (SP263) Assay (15). The TC ≥ 25% cut-off point was implemented for patient selection in the phase II HAWK and CONDOR trials (16, 17), and for stratification in the phase III EAGLE and KESTREL studies of R/M HNSCC (18, 19). In the HAWK trial, durvalumab-treated patients with PD-L1–high expression (TC ≥ 25%) had an ORR of 16.2%, a median duration of response of 10.3 months, and a median OS of 7.1 months (16). In the phase II CONDOR trial, patients with PD-L1–low/negative expression (TC < 25%), who were treated with durvalumab alone, had an ORR of 9.2% and median OS of 6.0 months (17). In the phase III EAGLE trial, data from the durvalumab monotherapy arm demonstrated a median OS of 9.8 months in patients with tumor PD-L1 expression ≥25%, compared with 7.6 months in patients with tumor PD-L1 expression <25% (18). Collectively, data from the second-line trials of durvalumab in R/M HNSCC suggest that there may be a more effective cut-off point for optimal identification of patients with survival benefit from durvalumab monotherapy.

Here, we present an analysis of a mature dataset with a substantial cohort of patients from the HAWK and CONDOR trials to evaluate different PD-L1 cut-off points in both ICs and TCs to develop an algorithm for optimal patient selection. The mature dataset from this study enabled the inclusion of PFS and OS as well as ORR in our analyses. These findings may help aid in identifying patients with HNSCC who may benefit from anti-programmed cell death-1/PD-L1 agents.

## Materials and Methods

### Data Sources

Durvalumab data from the phase II HAWK (*n* = 112) and CONDOR (*n* = 67) trials of patients with previously treated R/M HNSCC were included in the analyses (16, 17). The study designs of the HAWK and CONDOR have been published previously (16, 17). Briefly, HAWK was a single-arm phase II trial that evaluated durvalumab monotherapy in patients with PD-L1–high expression (TC ≥ 25%). CONDOR was an open-label, multicenter, global phase II trial in which patients with PD-L1–low/negative expression (TC < 25%) were randomized 1:1:2 to receive durvalumab, tremelimumab (anti-CTL-associated antigen 4), or durvalumab in combination with tremelimumab. Patients ≥18 years of age with histologically confirmed R/M HNSCC of the oral cavity, oropharynx, larynx, or hypopharynx not amenable to therapy with curative intent and with tumor progression or recurrence during or after treatment with only one systemic platinum-based regimen for R/M disease were included. PD-L1 expression for patient selection was assessed using the VENTANA PD-L1 (SP263) Assay (Roche, catalog no. 790-4905, RRID:AB_2819099; ref. 15). The studies were performed in accordance with ethical principles of the Declaration of Helsinki and are consistent with Good Clinical Practice guidelines of the International Conference on Harmonization. The study protocols were approved by local Institutional Review Boards. All patients provided written informed consent. The primary endpoint of each study was ORR using blinded independent central review as measured by RECIST, version 1.1. Secondary efficacy endpoints included PFS and OS.

### Cut-off Point Determination

Durvalumab efficacy data were pooled from the HAWK and CONDOR trials at the cut-off point date of March 31, 2017. TC PD-L1 expression was scored as the percentage of TCs with membrane staining at any intensity above the control. The scoring bins were <1%, ≥1%, ≥5%, ≥10%, and ≥20% for CONDOR samples and 25%, 30%, 40%, 50%, 60%, 70%, 75%, 80%, 90%, and 100% for HAWK samples (Supplementary Table S1). ICs with PD-L1 staining were expressed as a proportion of the ICs present (ICP) in the tumor area. When ICP was ≥1%, IC scores were estimated and scored as deciles and quartiles (i.e., 0%, 10%, 20%, 25%, 30%, 40%, 50%, 60%, 70%, 75%, 80%, 90%, 100%). In the rare cases that the ICP was <1%, PD-L1 IC expression was categorized as either 0%, <100%, or 100% (Supplementary Table S2). This rule was applied because it was deemed not possible to determine the percent staining when very few ICs were present. Data from subsequent cut-off dates on June 21, 2018 for HAWK and August 27, 2018 for CONDOR were used to confirm the choice of algorithm.

### Statistical Analyses

Regression analysis was used to assess the association between efficacy outcomes, with continuous PD-L1 expression (TC or IC) as covariates. Simple linear regression models were used to assess the association between maximum shrinkage in the sum of target lesions and PD-L1 expression, adjusted by baseline sum of target lesions, and the association between PD-L1 expression and the sum of target lesion size. In both models, the explanatory power of baseline PD-L1 expression on change in tumor volume was assessed using a prespecified threshold of *P* < 0.2 as a filter for significance.

Efficacy endpoints were ORR, change in the sum of target lesion size, PFS, and OS. Logistic regression was used to fit ORR with continuous PD-L1 expression as above, whereas univariate and multivariate Cox regression models were used for PFS and OS. In the multivariate Cox model, HRs were adjusted for age, gender, human papillomavirus (HPV) status (HPV positive vs. HPV negative), and smoking status—variables reported to impact PD-L1 status and/or efficacy outcomes (4, 20, 21). Cut-off points used were based on the exploratory binned scores provided by the pathologists in the studies, in addition to scoring above and below TC ≥ 25%. Best association required continuous PD-L1 score to be fitted into a linear model using the change in the sum of target lesion size as response, with baseline sum of target lesion size, relevant clinical endpoints, and biomarker status as covariates; this was repeated over a range of predefined cut-off points, with the final cut-off point selected on the basis of maximal statistical significance of the association.

The prevalence of PD-L1 (at TC ≥ 25%) in the HAWK and CONDOR screened patients (i.e., natural prevalence) was 27% (4). Because of the enrichment of TC ≥ 25% as a result of selection criteria in the HAWK study, the PD-L1 TC ≥ 25% prevalence in the pooled population was substantially higher (62%). It was not representative of an all-comers population. Thus, bootstrapping modeling of the OS HR was performed across the various combined TC/IC subgroups. All statistical analyses were conducted using SAS software, version 9.4 (SAS Institute).

### Assay Validation

Following the identification of the optimal cut-off point, the assay was validated for reader precision, inter-laboratory reproducibility, and tissue thickness.

#### Reader Precision

A cohort of 100 prescreened HNSCC cases [consisting of 50 PD-L1–high and 50 PD-L1–low/negative cases (including 10 borderlines)] were tested for reader precision. Three trained pathologists read the samples twice in a blinded and randomized fashion, with a washout period of at least 2 weeks between reads. The acceptance criteria were defined as a minimum of 85% average positive agreement (APA) and average negative agreement (ANA) for PD-L1–high or PD-L1–low/negative status across multiple readers (between-reader), and a minimum of 85% APA and ANA, and a minimum of 90% overall percentage agreement (OPA), within the same reader. The background/cross-reactivity was required not to interfere with slide interpretation in ≥90% of the tissue samples stained.

#### Inter-laboratory Reproducibility

A total of 28 cases [consisting of 14 PD-L1–high and 14 PD-L1–low/negative cases (including four borderlines)] were tested in three laboratories with two readers at each site for 5 non-consecutive days; thus, the expected total number of observations was 840 (28 × 5 × 3 × 2). The reproducibility of the VENTANA PD-L1 (SP263) Assay (Roche, catalog no. 790-4905, RRID:AB_2819099) results was determined by the OPA across all observations. The primary acceptance criterion was defined as an OPA of ≥85%.

#### Cut-slide Stability

Cut-slide stability was assessed on four tissue samples stained with the recommended assay protocol after storage at two slide storage conditions (2°C–8°C and 30°C) at various timepoints (day 0 to month 13). The acceptance criteria were defined as variation of staining intensity ≤1.0 and concordant PD-L1 status at various timepoints after sectioning with acceptable background staining in >90% samples. Day 0 served as the comparator for tissue samples stained with the recommended assay protocol.

#### Tissue Thickness

For assessment of tissue thickness, a total of four tissue samples were sectioned at thicknesses ranging 2–7 µm and stained using the recommended assay protocol. The acceptance criteria were defined as an OPA of ≥90%, where 4-µm-thick slides served as the comparator for tissue samples sectioned at various thicknesses. The non-specific background was required not to interfere with slide interpretation in ≥90% of the tissue samples stained.

### Data Availability Statement

Data underlying the findings described in this article may be obtained in accordance with AstraZeneca's data sharing policy described at: https://astrazenecagrouptrials.pharmacm.com/ST/Submission/Disclosure.

## Results

### Cut-off Point Determination

#### Efficacy of Durvalumab Monotherapy with Various PD-L1 Cut-off Points

An initial regression analysis suggested that PD-L1 expression on both TCs and ICs was associated with tumor shrinkage. We did not observe a correlation between TC and IC staining in pooled samples from durvalumab-treated patients (Supplementary Fig. S1).

Using pooled data from the HAWK (*n* = 112) and CONDOR (*n* = 67) trials, we assessed the association of PD-L1 expression with durvalumab efficacy. There was a general trend toward increasing median OS with increasing TC PD-L1 expression (Supplementary Fig. S2). The trend was less marked for PFS (Supplementary Fig. S3). The longest median OS (9.8 months [95% confidence interval (CI), 5.0–13.9]) and median PFS [3.4 months (95% CI, 1.9–5.4)] for TC PD-L1 expression were observed at the TC ≥ 50% cut-off point. There was an increase in median OS with increasing IC PD-L1 expression (Supplementary Fig. S2), and a trend toward increasing PFS (Supplementary Fig. S3). For IC PD-L1 expression, the longest median OS [10.2 months (95% CI, 5.0–not reached)] and median PFS [3.7 months (95% CI, 2.1–5.6)] were observed at the IC ≥ 25% cut-off point.

**FIGURE 1:**
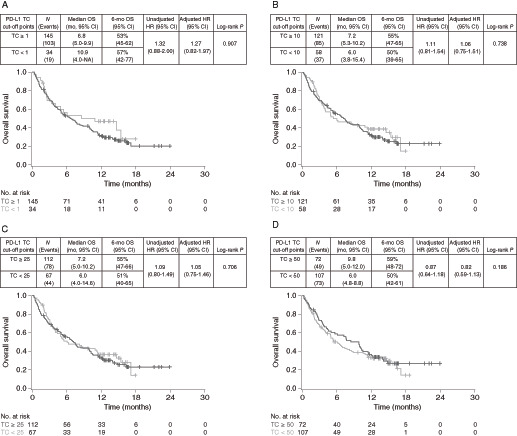
OS in PD-L1–high versus PD-L1–low/negative durvalumab-treated patients at different TC-based cut-off points. Kaplan–Meier plots of OS for cut-off points of TC1% (**A**), TC10% (**B**), TC25% (**C**), and TC50% (**D**). OS, overall survival; PD-L1, programmed cell death ligand-1; TC, tumor cell.

**FIGURE 2:**
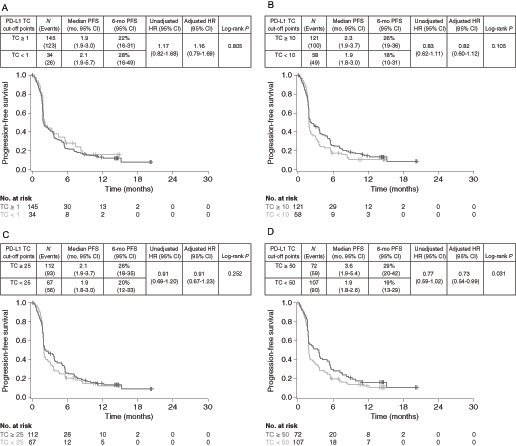
PFS in PD-L1–high versus PD-L1–low/negative durvalumab-treated patients at different TC-based cut-off points. Kaplan–Meier plots of PFS for cut-off points of TC1% (**A**), TC10% (**B**), TC25% (**C**), and TC50% (**D**). PD-L1, programmed cell death ligand-1; PFS, progression-free survival; TC, tumor cell.

**FIGURE 3:**
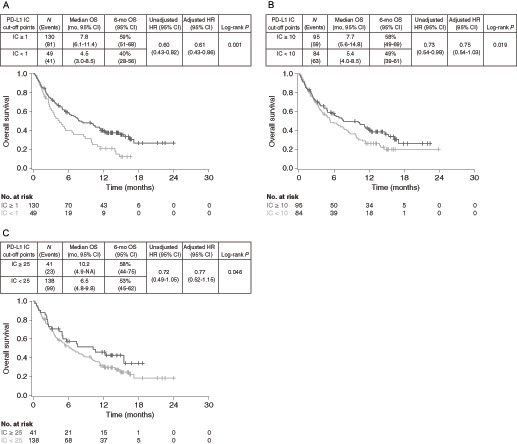
OS in PD-L1–high versus PD-L1–low/negative durvalumab-treated patients at different IC-based cut-off points. Kaplan–Meier plots of OS for cut-off points of IC1% (**A**), IC10% (**B**), and IC25% (**C**). IC, immune cell; NA, not available; OS, overall survival; PD-L1, programmed cell death ligand-1.

**FIGURE 4:**
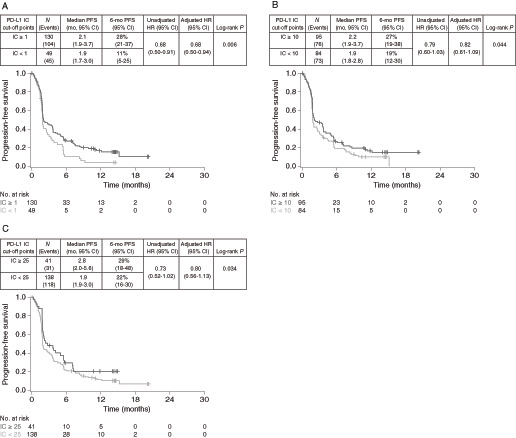
PFS in PD-L1–high versus PD-L1–low/negative durvalumab-treated patients at different IC-based cut-off points. Kaplan–Meier plots of PFS for cut-off points of IC1% (**A**), IC10% (**B**), and IC25% (**C**). IC, immune cell; PD-L1, programmed cell death ligand-1; PFS, progression-free survival.

The efficacy of TC PD-L1–high versus PD-L1–low/negative subgroups was compared at various cut-off points to evaluate their predictive value, that is, the ability of the biomarker cut-off point to distinguish between patients who benefit and those who do not (Figs. 1A–D and 2A–D). The greatest survival benefit was observed in patients with tumor PD-L1 expression TC ≥ 50 [OS HR 0.82 (95% CI, 0.59–1.13), PFS HR 0.73 (95% CI, 0.54–0.99)]. Taking the same approach for IC PD-L1 expression, the findings suggested that there was good differentiation between patients with PD-L1–high and PD-L1-low/negative expression at the ≥1%, ≥10%, and ≥25% cut-off points, indicating they were all suitable for combination with the TC ≥ 50% cut-off point (Figs. 3A–C and 4A–C).

**FIGURE 5:**
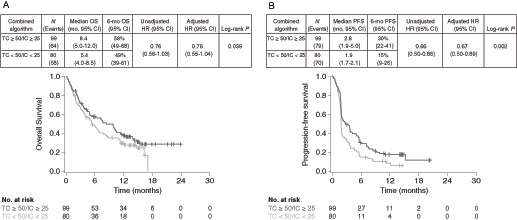
Kaplan–Meier plots for OS (**A**) and PFS (**B**) in durvalumab-treated patients using the combined TC50%/IC25% algorithm. OS, overall survival; PFS, progression-free survival; TC, tumor cell.

Finally, we evaluated the predictive value of algorithms combining the TC ≥ 50% cut-off point with IC ≥ 1%, ≥10%, and ≥25% cut-off points. When a combined algorithm of TC PD-L1 ≥ 50% or IC PD-L1 ≥ 25% (TC ≥ 50%/IC ≥ 25%) was used to select patients with PD-L1–high expression, compared with TC < 50%/IC < 25% for PD-L1–low/negative expression, higher ORR (17.2% vs. 8.8%; Table 1) and longer median OS (8.4 vs. 5.4 months) were observed (Fig. 5A). The difference in OS was associated with an unadjusted HR of 0.76 (90% CI, 0.56–1.03) and an adjusted HR of 0.76 (90% CI, 0.55–1.04; Fig. 5A). Median PFS was also longer for patients with PD-L1–high compared with PD-L1–low/negative expression (2.8 vs. 1.9 months), with unadjusted and adjusted PFS HRs of 0.66 (90% CI, 0.50–0.86) and 0.67 (90% CI, 0.50–0.89), respectively (Fig. 5B).

**TABLE 1:** ORR at different cut-off points in durvalumab-treated patients.

Cut-off point	ORR, % (95% CI) PD-L1–high	ORR, % (95% CI) PD-L1–low/negative
**TC1%**	14.5 (9.2–21.3)	8.8 (1.9–23.7)
**TC10%**	15.7 (9.7–23.4)	8.6 (2.9–19.0)
**TC25%**	16.1 (9.8–24.2)	9.0 (3.4–18.5)
**TC50%**	16.7 (8.9–27.3)	11.2 (5.9–18.8)
**IC1%**	16.2 (10.3–23.6)	6.1 (1.3–16.9)
**IC10%**	15.8 (9.1–24.7)	10.7 (5.0–19.4)
**IC25%**	17.1 (7.2–32.1)	12.3 (7.3–19.0)
**T50%/IC25%**	17.2 (10.3–26.1)	8.8 (3.6–17.2)

Abbreviations: CI, confidence interval; IC, immune cell; ORR, objective response rate; PD-L1, programmed cell death ligand-1; PFS, progression-free survival; TC, tumor cell.

Results of a bootstrap analysis evaluating different combined algorithms and modeling for an all-comer population showed that the TC ≥ 50%/IC ≥ 25% algorithm was optimal with the lowest OS HR of 0.82 (90% CI, 0.53–1.20; Supplementary Fig. S4). Therefore, the optimal combined algorithm was determined to be TC ≥ 50%/IC ≥ 25%. Data from later cut-off dates of June 21, 2018 and August 27, 2018 for HAWK and CONDOR, respectively, were used to confirm the selection of the TC ≥ 50%/IC ≥ 25% algorithm. A comparison of PD-L1–high versus PD-L1–low/negative at a cut-off point of TC ≥50%/IC ≥ 25% yielded an adjusted OS HR of 0.71 (90% CI, 0.53–0.94) and an adjusted PFS HR of 0.69 (90% CI, 0.52–0.91; Supplementary Fig. S5). These values are consistent with the earlier data analysis.

### Assay Validation

#### Formal Definition of Algorithm

PD-L1 status was determined by the percentage of TCs with any membrane staining above background or by the percentage of tumor-associated ICs with staining (IC+) at any intensity above background. Thus, based on the VENTANA PD-L1 (SP263) Assay scoring algorithm and the determined optimal cut-off points, PD-L1 status was considered high if ≥50% of TCs exhibit membrane staining; or ICP > 1% and IC+ ≥ 25%; or ICP = 1% and IC+ = 100%. PD-L1 status was considered low/negative if none of the criteria for PD-L1–high status were met (Supplementary Table S2).

#### Reader Precision

APA and ANA between the three readers were both 98.0%. The within-reader APA, ANA, and OPA were all 98.7%. The background staining was 100% acceptable. These findings met the prespecified acceptance criteria for the assay (Supplementary Table S3).

#### Inter-laboratory Reproducibility

The positive percent agreement and negative percent agreement for the inter-laboratory reproducibility were 99.0% and 98.1%, respectively. OPA was 98.6% and met the prespecified acceptance criteria for the assay (Supplementary Table S3).

#### Cut-slide Stability

The staining performance for tissues stored at 2°C–8°C and 30°C for up to 9 months were consistent with results achieved on day 0 and met the prespecified acceptance criteria (Supplementary Table S3). Because of a decreasing trend in PD-L1 expression, the recommended cut-slide stability dating was set to 7 months.

#### Tissue Thickness

Appropriate antibody staining was achieved across all tissue section thickness tested (2, 3, 4, 5, 6, and 7 µm) with acceptable background staining and met the prespecified acceptance criteria (Supplementary Table S3). The recommended tissue thickness is 4–5 µm.

## Discussion

Durvalumab has shown antitumor activity in patients with R/M HNSCC (16–18). However, as with all ICIs, many patients do not respond to treatment, highlighting the importance of refining strategies for patient selection (22). Using the VENTANA PD-L1 (SP263) Assay, the TC25% cut-off point was originally used to select or randomize patients for treatment with durvalumab in clinical studies (<25% for CONDOR and ≥25% for HAWK and EAGLE; refs. 16–18). The current study findings establish that the higher cut-off point of TC ≥ 50% is associated with better survival outcomes compared with other TC cut-off points (≥1%, ≥10%, and ≥25%) when applied to the VENTANA PD-L1 (SP263) Assay. This observation could be anticipated given that PD-L1 expression is a continuum rather than being binary (23). Perhaps more surprising is the substantial association of IC PD-L1 expression with survival outcomes. At a cut-off point of IC1%, the adjusted HR was 0.61 for the comparison of median OS between IC ≥ 1% and IC < 1%. Median OS was 7.8 months with IC ≥ 1%, but was longer at 10.2 months with IC ≥ 25%. Overall, the results of our analyses show that, while PD-L1 expression on TCs is a useful biomarker, PD-L1 expression on ICs is similarly important in enriching for responses.

The results from our assessment of PD-L1 expression on ICs alone indicate that a low cut-off point of IC ≥ 1% is the most discriminatory in terms of survival for patients with PD-L1–high expression versus those with PD-L1–low/negative expression. However, of the four most feasible TC/IC algorithm combinations evaluated, the bootstrapped survival analysis indicated that TC ≥ 50%/IC ≥ 25% was optimal. Moreover, any assay developed to select patients for ICI treatment must be highly reproducible across laboratories and between pathologists. Scoring at low cut-off points can present a challenge. On the basis of experience with developing a similar method of scoring PD-L1 on ICs in metastatic urothelial cancer, IC ≥ 25% was considered to be more reproducible (i.e., higher within-reader precision) than IC ≥ 1% or IC ≥ 10%, and, thus, would provide a more robust diagnostic assay for clinical use (6). Our analytic studies confirmed the TC ≥ 50%/IC ≥ 25% cut-off point could be reproducibly scored in HNSCC samples.

Our method of scoring PD-L1 IC expression is determined as a percentage of the total ICs present. Other methods of scoring ICs assess the proportion of PD-L1–expressing ICs in the tumor area, so that the degree of IC infiltration is a contributing factor to the results (5). There is evidence that the extent of tumor-infiltrating lymphocytes is an independent prognostic factor for disease-free survival in patients with HNSCC (24), which may be a measure of the immune response or immune competency of the patient. An analysis by Kim and colleagues (25) showed that PD-L1–positive tumor-infiltrating lymphocytes were strongly associated with a favorable prognosis in patients with surgically resected HNSCC and that high PD-L1 expression on ICs, but not TCs, was an independent favorable prognostic factor for recurrence-free survival and OS.

It is established that patients with HPV-positive oropharyngeal cancer have significantly better survival outcomes than patients with HPV-negative oropharyngeal cancer (20). Tumors from patients with HPV-positive oropharyngeal cancer have been shown to have a higher frequency of PD-L1–expressing ICs than those from patients with HPV-negative oropharyngeal cancer, concomitant with a significantly higher 5-year OS rate (25). We used an adjusted HR that included HPV status in our analyses, yet, without a comparator arm, we cannot be certain whether ICs are predictive or merely prognostic. Thus, by using PD-L1 expression on ICs as a selection biomarker, there is a risk that patients will derive no additional benefit from ICIs over chemotherapy, that is, the marker is prognostic but not predictive. However, this risk may be offset through the retention of the TC component in the assay.

PD-L1 expression in TCs at different cut-off points, measured using the VENTANA PD-L1 (SP263) Assay, was reported to not be prognostic for OS in patients with HNSCC who received standard-of-care chemotherapies (26). Data from studies of pembrolizumab in HNSCC have shown that, compared with tumor proportion score (defined as the percentage of TCs with complete or partial membrane staining at any intensity), a combined positive score (CPS; the number of PD-L1–stained TCs and ICs relative to the total number of all TCs, with ICs including lymphocytes and macrophages) had a superior predictive value (12, 13, 27). In the KEYNOTE-040 phase III trial of previously treated patients with R/M HNSCC who received pembrolizumab or chemotherapy plus cetuximab, the combined TC and IC algorithm of CPS ≥ 1 was predictive, but not prognostic, of survival (27). Furthermore, in the KEYNOTE-048 trial of previously untreated patients with R/M HNSCC, an increase in cut-off point from CPS1 to CPS20 increased the 12-month OS rate in patients treated with pembrolizumab without substantial impact on OS in the chemotherapy plus cetuximab arm (2).

In our analyses, we did not identify any relationship between PD-L1 expression on TCs and ICs. This finding is consistent with the results of Kim and colleagues (25), which showed an overlap of only 0.2% between PD-L1 expression in TCs and ICs when assessed separately at TC ≥ 50% and IC ≥ 50%. Using transcriptome analyses, these investigators showed much higher levels of effector T-cell markers, such as IFNγ, in the IC ≥ 50% subgroup compared with the TC ≥ 50% subgroup. These results suggest that PD-L1 expression is regulated independently in TCs and ICs, which, in the latter, may be mediated by adaptive mechanisms that reflect preexisting immunity. Similar results have been observed in other tumor types, such as non–small cell lung cancer, in which PD-L1 expression on ICs was found to be regulated by adaptive IFNγ-mediated mechanisms associated with increased tumor-infiltrating lymphocytes and effector T cells (28). Collectively, the evidence supports a role for PD-L1 expression in both TCs and ICs in attenuating antitumor immune responses, thus providing the rationale for measuring PD-L1 expression on both components of the tumor microenvironment.

We determined that TC ≥ 50%/IC ≥ 25% was the optimal PD-L1 algorithm for identifying patients with HNSCC most likely to benefit from durvalumab treatment. The challenges associated with PD-L1 as a biomarker are the imperfect association of response and that PD-L1 expression is a continuum. Thus, setting the cut-off point too high would exclude some patients who would benefit from ICI therapy, whereas setting the cut-off point too low would include some patients who may not respond to ICI therapy. Orthogonal methods could be used to assist clinicians in identifying patients with HNSCC who would most benefit from ICI therapies, such as neutrophil-to-lymphocyte ratio (29) and tumor mutational burden (30).

PD-L1 expression in both TCs and ICs has been shown to be predictive of ICI efficacy in other tumor types, including metastatic urothelial carcinoma and advanced esophageal cancer (31–33). In a phase I/II study of previously treated patients with metastatic urothelial carcinoma, using the VENTANA PD-L1 (SP263) Assay, a combined TC ≥ 25%/IC ≥ 25% algorithm was superior to other algorithms at predicting response to durvalumab (31). Using the same assay and scoring algorithm in the phase III DANUBE trial of previously untreated patients, those who had high tumor PD-L1 expression survived longer than the all-comer population when treated with durvalumab or durvalumab plus tremelimumab (32). The algorithm was also transferable to another anti-PD-L1 agent, avelumab, where PD-L1 expression, assessed by the assay, was predictive of survival in the JAVELIN Bladder 100 maintenance study of patients with metastatic urothelial carcinoma who had received first-line, platinum-based chemotherapy (34). On the basis of data from first- and second-line studies in metastatic urothelial carcinoma, and given that a survival benefit was observed in KEYNOTE-040 and KEYNOTE-048 at CPS ≥ 1, the results suggest that a combined TC/IC algorithm may be suitable in both the first- and second-line settings for a given tumor type.

A limitation of our analyses is that the cut-off point determination was based on durvalumab data from studies without a comparator arm. Thus, validation of the prognostic versus predictive value of the TC ≥ 50%/IC ≥ 25% algorithm determined in this study is planned within the randomized, controlled, phase III KESTREL study of first-line R/M HNSCC (19). Another limitation of our analyses was the inability to determine the optimal cut-off point for selecting patients to receive treatment with the combination of durvalumab and tremelimumab. This was due to the fact that only patients with low tumor PD-L1 expression (TC < 25%) were randomized in the CONDOR phase II trial, which would not have represented a natural population. Interestingly, the scoring algorithm developed using data for durvalumab monotherapy in second-line studies of metastatic urothelial carcinoma was highly effective for durvalumab plus tremelimumab in the DANUBE trial, and raises the possibility that the same may be true in HNSCC. We showed that IC ≥ 25% was a useful cut-off point for predicting survival outcomes with the combination. However, larger, randomized controlled trials will be required to determine whether the TC ≥ 50%/IC ≥ 25% algorithm can also predict response to durvalumab plus tremelimumab in R/M HNSCC.

In this investigation, we established that the cut-off point of TC ≥ 50% improved survival outcomes compared with lower TC cut-off points, including TC ≥ 25%, which was used to select patients for treatment or randomization in prior studies of HNSCC. However, increasing evidence suggests that expression of PD-L1 in both TCs and ICs may be predictive of response to PD-L1 inhibitors such as durvalumab, and findings from the current study confirm that both TC and IC PD-L1 expression have predictive value for efficacy outcomes in patients with R/M HNSCC. Using the VENTANA PD-L1 (SP263) Assay, the combined TC ≥ 50%/IC ≥ 25% algorithm was associated with increased durvalumab efficacy in R/M HNSCC and discrimination of patients with survival benefit from durvalumab treatment. Analytic data show that the TC ≥ 50%/IC ≥ 25% cut-off point algorithm can be scored reproducibly in HNSCC, providing a practical, robust assay for patient selection.

## Supplementary Material

Supplementary Data Supplemental Material
