# Supplementary material for: Association of PD-L1 Expression on Tumor and Immune Cells with Survival in Recurrent or Metastatic Head and Neck Squamous Cell Carcinoma and Assay Validation
Source: Cancer Res Commun. 2022 Jan 20;2(1):39–48. doi: 10.1158/2767-9764.CRC-21-0032 (PMC9973403; doi:10.1158/2767-9764.CRC-21-0032)
Supplement: Supplementary Data — Supplemental Material [file crc-21-0032-s01.pdf]

Supplementary Fig. S1. Assessment of the correlation between TC and IC staining in samples from durvalumab-treated patients.

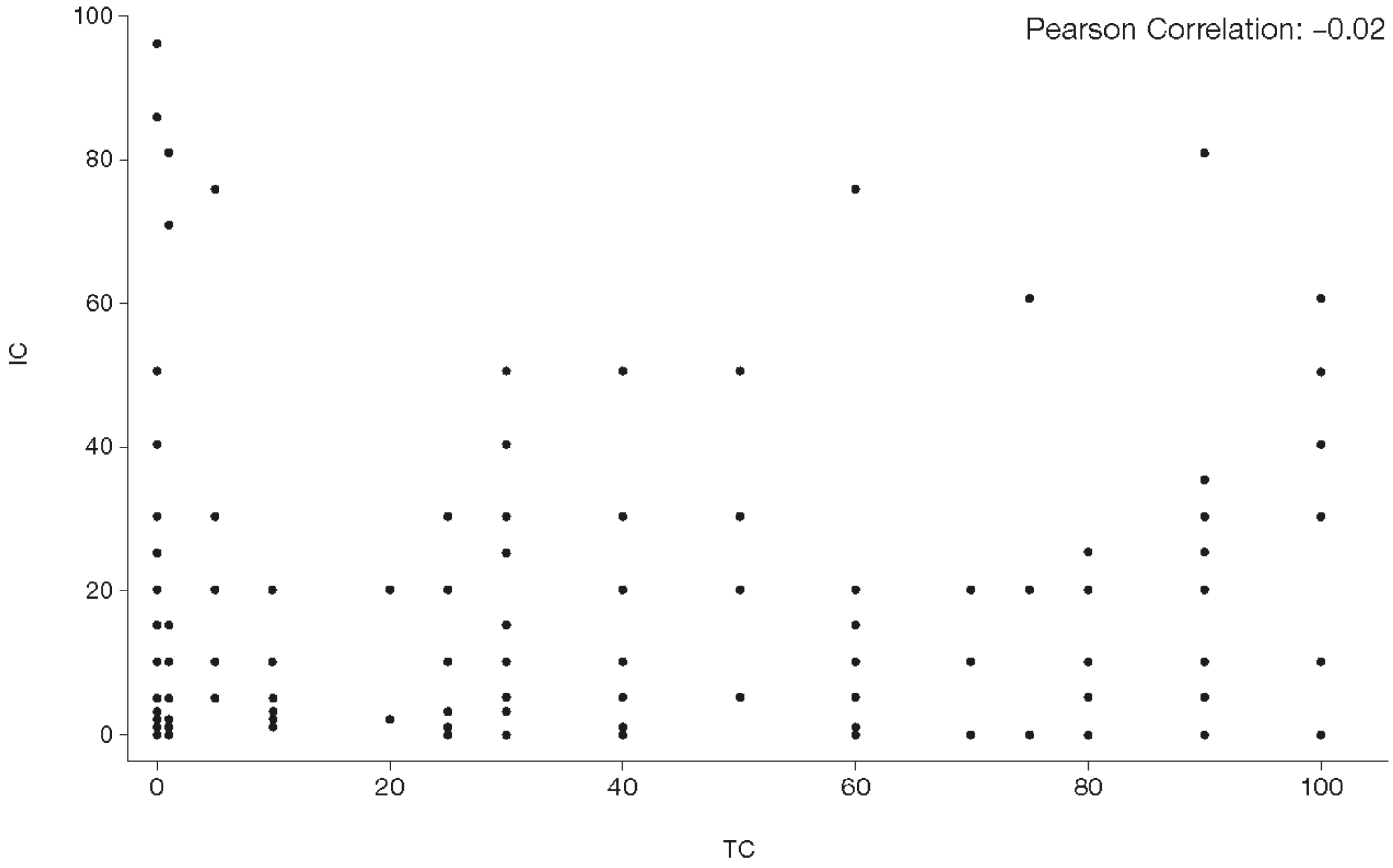

IC, immune cell; TC, tumor cell.

Supplementary Fig. S2. Kaplan–Meier plots of OS in durvalumab-treated patients at a range of PD-L1 (A) TC cutpoints and (B) IC cutpoints. These plots are based on the original PD-L1 scoring data with only 18 months of survival follow-up.

(A)

PD-L1 TC Cutpoints	*N* (Events)	Median OS (mo, 95% CI)	6-mo OS (95% CI)
TC ≥50	72 (40)	9.8 (5.0-13.9)	59% (48-71)
TC ≥25	111 (65)	6.9 (5.0-9.9)	55% (46-65)
TC ≥10	120 (71)	6.9 (5.3-9.9)	55% (47-65)
TC ≥1	142 (87)	6.6 (5.0-9.8)	53% (45-62)
TC = 0	35 (16)	NA (3.3-NA)	55% (40-75)
All cutpoints	179 (103)	6.8 (5.0-9.9)	54% (47-62)

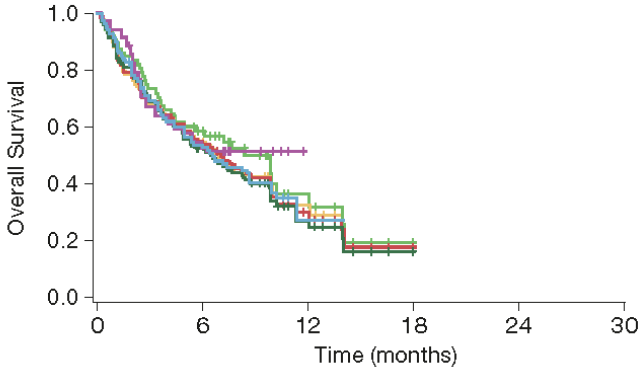

No. at risk

All	179	62	11	0	0	0
TC = 0	35	16	0	0	0	0
TC ≥1	142	66	11	0	0	0
TC ≥10	120	57	10	0	0	0
TC ≥25	111	52	10	0	0	0
TC ≥50	72	36	8	0	0	0

(B)

PD-L1 IC Cutpoints	*N* (Events)	Median OS (mo, 95% CI)	6-mo OS (95% CI)
IC ≥50	17 (8)	NA (3.0-NA)	52% (32-83)
IC ≥25	42 (19)	10.2 (5.0-NA)	59% (45-76)
IC ≥10	94 (49)	7.7 (5.0-NA)	58% (48-69)
IC ≥1	131 (69)	7.7 (5.6-11.3)	58% (50-67)
IC = 0	47 (34)	4.5 (2.8-9.9)	42% (30-59)
All cutpoints	179 (103)	6.8 (5.0-9.9)	54% (47-62)

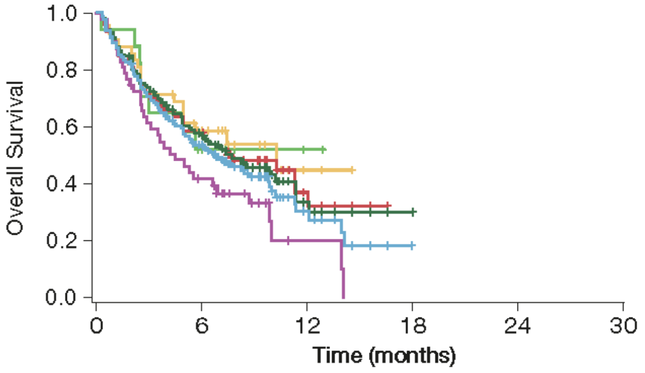

No. at risk

All	179	62	11	0	0	0
IC = 0	47	18	2	0	0	0
IC ≥1	131	64	9	0	0	0
IC ≥10	94	47	8	0	0	0
IC ≥25	42	20	2	0	0	0
IC ≥50	17	6	1	0	0	0

IC, immune cell; OS, overall survival; PD-L1, programmed cell death ligand-1; TC, tumor cell.

Supplementary Fig. S3. Kaplan–Meier plots of PFS in durvalumab-treated patients using a range of PD-L1 (A) TC cutpoints and (B) IC cutpoints. These plots are based on the original PD-L1 scoring data with only 18 months of survival follow-up.

(A)

PD-L1 TC Cutpoints	*N* (Events)	Median PFS (mo, 95% CI)	6-mo PFS (95% CI)
TC ≥50	72 (56)	3.4 (1.9-5.4)	27% (18-40)
TC ≥25	111 (87)	2.0 (1.9-3.7)	24% (17-34)
TC ≥10	120 (94)	2.1 (1.9-3.7)	25% (18-34)
TC ≥1	142 (115)	1.9 (1.8-3.0)	21% (15-30)
TC = 0	35 (26)	2.1 (1.9-5.6)	23% (12-44)
All cutpoints	179 (143)	2.0 (1.9-3.0)	21% (16-29)

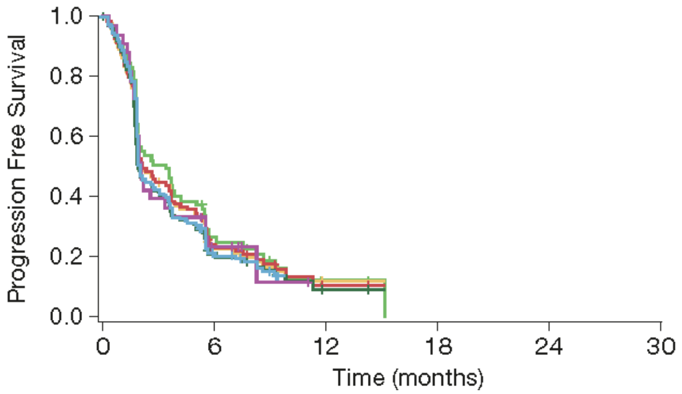

No. at risk

All	179	27	5	0	0	0
TC = 0	35	5	0	0	0	0
TC ≥1	142	22	3	0	0	0
TC ≥10	120	21	3	0	0	0
TC ≥25	111	19	3	0	0	0
TC ≥50	72	14	3	0	0	0

(B)

PD-L1 IC Cutpoints	*N* (Events)	Median PFS (mo, 95% CI)	6-mo PFS (95% CI)
IC ≥50	17 (12)	2.2 (1.8-NA)	25% (10-61)
IC ≥25	42 (28)	3.7 (2.1-5.6)	30% (18-50)
IC ≥10	94 (70)	2.2 (1.9-3.6)	27% (19-38)
IC ≥1	131 (99)	2.1 (1.9-3.6)	26% (19-35)
IC = 0	47 (43)	1.9 (1.7-2.7)	11% (5-25)
All cutpoints	179 (143)	2.0 (1.9-3.0)	21% (16-29)

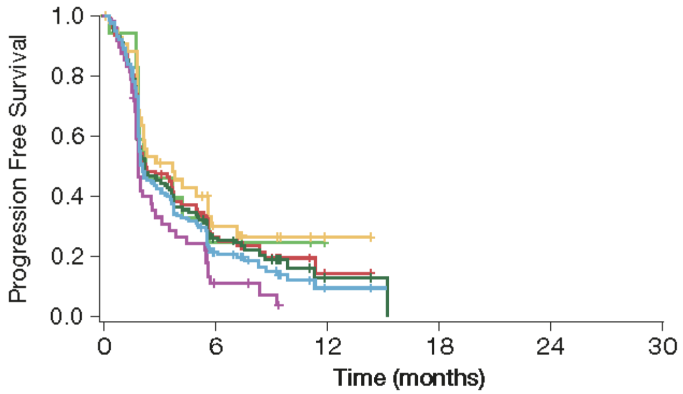

No. at risk

All	179	27	5	0	0	0
IC = 0	47	4	0	0	0	0
IC ≥1	131	23	3	0	0	0
IC ≥10	94	18	2	0	0	0
IC ≥25	42	8	1	0	0	0
IC ≥50	17	3	0	0	0	0

IC, immune cell; PD-L1, programmed cell death ligand-1; PFS, progression-free survival; TC, tumor cell.

Supplementary Fig. S4. Bootstrapped OS HR for HAWK and CONDOR combined data for durvalumab monotherapy (*n* = 190 patients). Data shows overall survival HR [BM+ vs. BM–] unadjusted Cox PH (with Ties handling method=Effron) highlighting optimal cutpoint of TC≥50 or IC≥25% with the lowest HR.

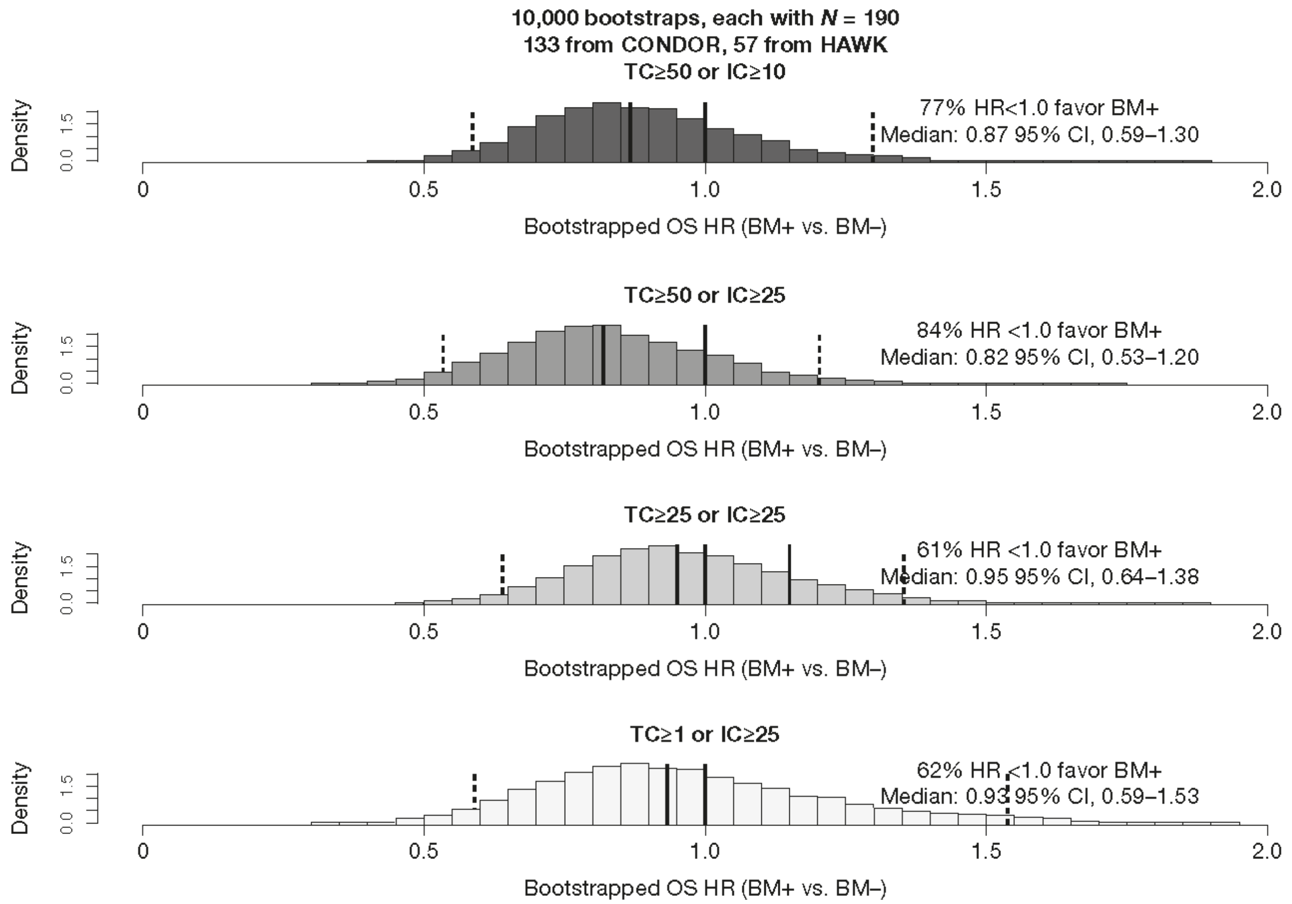

BM, biomarker; CI, confidence interval; HR, hazard ratio; IC, immune cell; OS, overall survival; TC, tumor cell.

Supplementary Fig. S5. Kaplan–Meier plots of (A) OS and (B) PFS using the TC50%/IC25% algorithm, based on updated data cutoffs for CONDOR (2018-08-27) and HAWK (2018-06-21).

(A)

Combined algorithm	*N* (Events)	Median OS (mo, 95% CI)	6-mo OS (95% CI)	Unadjusted HR (95% CI)	Adjusted HR (95% CI)
TC ≥50/IC ≥25	99 (80)	9.8 (5.0-11.5)	58% (47-67)	0.68 (0.52-0.90)	0.71 (0.53-1.04)
TC <50/IC <25	80 (71)	5.5 (3.8-8.3)	49% (37-59)		

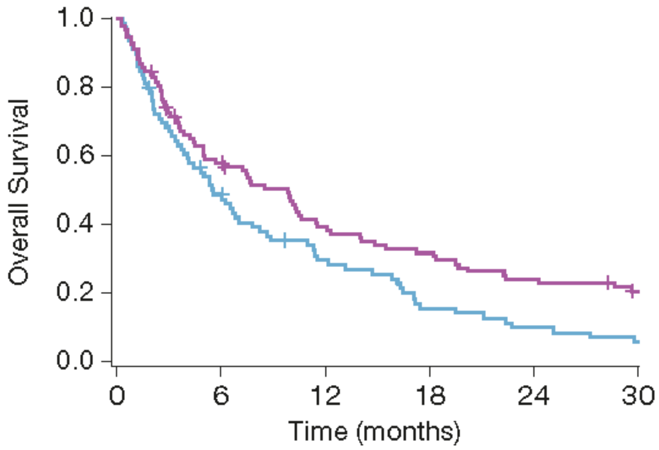

No. at risk						
TC ≥50/IC ≥25	99	55	36	29	22	16
TC <50/IC <25	80	37	21	11	7	4

(B)

Combined algorithm	*N* (Events)	Median PFS (mo, 95% CI)	6-mo PFS (95% CI)	Unadjusted HR (95% CI)	Adjusted HR (95% CI)
TC ≥50/IC ≥25	99 (89)	2.8 (2.0-5.0)	18% (11-27)	0.68 (0.52-0.88)	0.69 (0.52-0.91)
TC <50/IC <25	80 (74)	1.9 (1.8-2.1)	9% (4-19)		

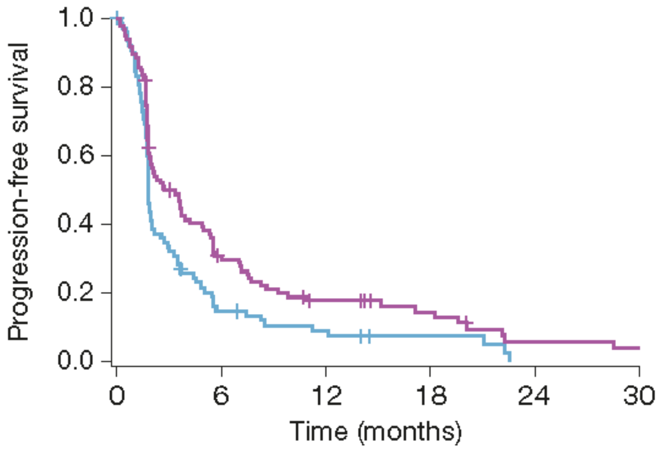

No. at risk						
TC ≥50/IC ≥25	99	28	14	9	3	2
TC <50/IC <25	80	11	6	3	0	0

CI, confidence interval; HR, hazard ratio; IC, immune cell; OS, overall survival; PFS, progression-free survival; TC, tumor cell.

Supplementary Table S1. TC PD-L1 expression levels according to HAWK and CONDOR categories.

CONDOR		HAWK	
**Scoring bin**	**Bin contents**	**Scoring bin**	**Bin contents**
<1%	<1%	25%	25%
≥1%	1–4%	30%	26–34%
≥5%	5–9%	40%	35–44%
≥10%	10–19%	50%	45–54%
≥20%	20–24%	60%	55–64%
		70%	65–74%
		75%	75%
		80%	76–84%
		90%	85–94%
		100%	95–100%

PD-L1, programmed cell death ligand-1; TC, tumor cell.

Supplementary Table S2. VENTANA PD-L1 (SP263) assay scoring algorithm for HNSCC.

**VENTANA PD-L1 (SP263) assay scoring algorithm for HNSCC**	
**PD-L1 interpretation**	**Staining description**
PD-L1 status is determined by the percentage of TCs with any membrane staining above background or by the percentage of tumor-associated immune cells (ICs) with staining (IC+) at any intensity above background. The percent of tumor area occupied by any tumor-associated ICs (ICs present; ICP) is used to determine IC+, which is the percent area of ICP exhibiting PD-L1 positive immune cell staining	
High status	PD-L1 status is considered high if any of the following are met:<br>▪ ≥50% of TCs exhibit membrane staining; or,<br>▪ ICP >1% and IC+ ≥25%; or,<br>▪ ICP = 1% and IC+ = 100%
Low/negative status	PD-L1 status is considered low/negative if:<br>▪ None of the criteria for PD-L1-high status are met

IC, immune cell; ICP, ICs present; PD-L1, programmed cell death ligand-1; HNSCC, head and neck squamous cell carcinoma; TC, tumor cell.

Supplementary Table S3. Design verification study results and analytical validation (interlaboratory reproducibility) at the TC≥50%/IC≥25% cutpoint.

Study outline	Design	Results	
Reader precision	Cohort previously screened for PD-L1 status; consisted of 100 tissue samples (50 PD-L1-high and 50 PD-L1-low/negative)	Between reader,<br>% (95% CI):<br>APA 98.0% (95.4–100.0)<br>ANA 98.0% (95.4–100.0)<br>OPA 98.0% (95.3–100.0)	Within reader,<br>% (95% CI):<br>APA 98.7% (97.1–99.7)<br>ANA 98.7% (97.1–99.7)<br>OPA 98.7% (97.3–99.7)
Interlaboratory reproducibility	Tested in three laboratories with two readers at each site for 5 non-consecutive days; 28 tissue samples enrolled (14 PD-L1-high and 14 PD-L1-low/negative)	Overall, % (95% CI)<br>PPA 99.0% (97.9–100.0)<br>NPA 98.1% (98.0–98.1)<br>OPA 98.6% (98.0–99.0)	
Cut-slide stability	Four tissue samples sectioned at 4 µm and stored at 2–8°C and 30°C for up to 13 months	Staining results at different storage temperatures and time points up to Month 9 were consistent with results achieved on Day 0. The recommended dating is 7 months.	
Tissue thickness	Four tissue samples sectioned at various thickness (2, 3, 4, 5, 6, 7 µm)	Demonstrated appropriate antibody staining across all thickness tested. The recommend tissue thickness is 4–5 µm.	

ANA, average negative agreement; APA, average positive agreement;CI, confidence interval; IC, immune cell; NPA, negative percent agreement; OPA, overall percent agreement; PD-L1, programmed cell death ligand-1; PPA, positive percent agreement; TC, tumor cell.
